# Cannabidiol Induces Autophagy to Protects Neural Cells From Mitochondrial Dysfunction by Upregulating SIRT1 to Inhibits NF-κB and NOTCH Pathways

**DOI:** 10.3389/fncel.2021.654340

**Published:** 2021-03-30

**Authors:** Shaolei Kang, Jinglin Li, Zhihui Yao, Jiaxin Liu

**Affiliations:** ^1^Medical School, Kunming University of Science and Technology, Kunming, China; ^2^Department of Radiology, The First Affiliated Hospital of Kunming Medical University, Kunming, China; ^3^Department of Burn and Plastic Surgery, 926 Hospital of People’s Liberation Army, Kaiyuan, China

**Keywords:** Cannabidiol, SIRT1, NF-κB, NOTCH, PD, SH-SY5Y cells

## Abstract

The protective effect of Cannabidiol on Parkinson’s disease (PD) has been found in recent study. However, the specific mechanism of the protective effect of Cannabidiol on PD nerve damage require further exploration. This study aims to investigate effect of Cannabidiol on MMP-induced Neural Cells (SH-SY5Y) mitochondrial dysfunction. MMP^+^ and Cannabidiol were used to treat SH-SY5Y cells, the cells viability was measured by MTT assay. The expression of Tyrosine hydroxylase (TH) in cells was measured by western blotting and Immunofluorescence staining. The relationship among Cannabidiol, Silent mating type information regulation 2 homolog-1 (SIRT1) and NOTCH signaling, NF-κB signaling was examined by western blotting. The effect of Cannabidiol on MMP^+^-induced mitochondrial dysfunction of SH-SY5Y cells was measured by western blotting. Cannabidiol alleviated loss of TH expression and cytotoxicity in the MPP^+^-induced SH-SY5Y cells. Further mechanistic investigation showed that Cannabidiol induced SH-SY5Y cells autophagy to protects cells from mitochondrial dysfunction by upregulating SIRT1 to Inhibits NF-κB and NOTCH Pathways. Taken together, Cannabidiol acts as a protector in PD.

## Introduction

Parkinson’s disease (PD) is a common neurodegenerative disease (Jasmin et al., 2020). More than 6 million individuals suffering from PD globally, which seriously endangers human health in their lives (Nobre et al., 2020). Pathologically, the characteristics of PD include the gradual loss of dopaminergic neurons in substantial nigra pars compacta and the abnormal accumulation of α-synuclein, which further leads to motor dysfunction (Sveinbjornsdottir, 2016; Opara et al., 2017). Currently, the common treatment for PD is the administration of a dopamine precursor to relieve the clinical symptoms of PD patients (Santiago et al., 2017). However, there are still no effective drugs or method to restore and prevent neuronal damage. Therefore, the development of new adjuvant therapies and therapeutic targets is crucial.

Cannabidiol is a component extracted of non-psychoactive natural from cannabis, It has antioxidant and anti-inflammatory properties (Atalay et al., 2019). A series of studies have confirmed that Cannabidiol are effective against Neurodegenerative disease, such as PD (Santos et al., 2015), AD (Watt and Karl, 2017). Although current studies have confirmed that Cannabidiol plays an active role in neurological diseases through G protein-coupled receptors, TRPV1 ion channels, and peroxisome proliferator-activated receptors (Santos et al., 2015), the specific protective mechanism of Cannabidiol on PD nerve damage has not been fully elucidated.

Insufficiency of mitochondria leads to mitochondrial dysfunction, contributing to the progression of PD characterized by dysfunctional energy metabolism (Wang et al., 2016). Previous studies have shown that mitochondrial dysfunction is related to the pathogenesis of PD progression. Moreover, the regulation of autophagy may be an important therapeutic strategy in PD, When mitochondrial damage causes mitochondrial dysfunction in nerve cells, it further leads to the development of PD. Therefore, promoting damaged mitochondrial autophagy can remove the aggregates of α-synuclein and other abnormal proteins, and protect nerve cells from mitochondrial dysfunction (Ho et al., 2020). In addition, Cannabidiol protects cell survival by improving mitochondrial dysfunction (Ammal Kaidery et al., 2019). However, the specific mechanism of Cannabidiol on improving mitochondrial dysfunction and autophagy is still unclear.

In this study, we exposed SH-SY5Y cells to MPP^+^ to mimic a cellular model of PD. simultaneously, the protective effect of Cannabidiol and its possible molecular mechanism were investigated through this model. We found that SIRT1 is closely related to the protection of SH-SY5Y cells from mitochondrial dysfunction by Cannabidiol. Further analysis showed that Cannabidiol induces autophagy to protect SH-SY5Y cells from mitochondrial dysfunction by upregulating SIRT1 to inhibits NF-κB and NOTCH pathways.

## Materials and Methods

### Materials

Cannabidiol (purity > 98%)were purchased from Biopurify (Chengdu, China). 3-(4,5-dimethyl-2-thiazolyl)-2, 5-diphenyltetrazolium bromide (MTT) and 1-Methyl-4-phenylpyridine (MPP^+^) were purchased from Sigma-Aldrich (St. Louis, MO, United States); Dulbecco’s modified Eagle medium and 10% fetal bovine serum (FBS) were purchased from Gibco (Grand Island, NY, United States). penicillin and streptomycin were purchased from Sigma-Aldrich (St. Louis, MO, United States). Primary antibodies against TH, SIRT1, α-synuclein, p62, LC3-I/II, Atg5/7, Parkin, PINK-1, Nrf2, SOD-1, DJ-1, GSH, NOTCH1, Hes1, p-p65, p65, p-Ikb, Ikb, and GAPDH were purchased from Cell Signaling Technology (Danvers, MA, United States).

### Cell Culture and Treatments

The SH-SY5Y human neuroblastoma cell line was purchased from Cell Bank, Shanghai Institutes for Biological Sciences (Shanghai, China), Cells were cultured in the DMEM with 10% fetal bovine serum, 100 U/ml penicillin and 100 μg/ml streptomycin (Sigma, United States) at 37°C in a 5% CO_2_ humidified incubator. The medium is replaced every 2 days. When the monolayer cell confluence reaches 75% or more, the cells are subculture. SH-SY5Y cells were pretreated with different concentrations of Cannabidiol for 24 h followed by incubation with MPP^+^ for another 24 h to observe the effects of Cannabidiol on autophagy in MPP^+^-induced cells.

### Western Blotting

The whole-cell proteins were extracted from the SH-SY5Y cells lysed in RIPA lysis buffer (Invitrogen, United States), and the protein concentrations were determined assessed using Bradford protein assay (Invitrogen, United States). After boiling and denaturation, Protein samples were separated by a 10% SDS-PAGE and transferred onto PVDF membranes. Then, the membranes were blocked with 50 g/L skim milk for 4 h at room temperature and incubated with primary antibodies (TH, SIRT1, α-synuclein, p62, LC3-I/II, Atg5/7, Parkin, PINK-1, Nrf2, SOD-1, DJ-1, GSH, NOTCH1, Hes1, p-p65, p65, p-Ikb, Ikb, and GAPDH) at 4°C overnight. The next day, the membranes were washed three times with PBS and incubated with secondary antibodies at room temperature for 1 h. The membranes were rinsed with TBST buffer and visualized using an enhanced chemiluminescence kit (Bio-Rad Laboratories, Inc). Finally, the protein bands were quantified using ImageJ software (NIH, Bethesda, MD, United States).

### MTT Assay

Cell viability was determined using the MTT assay. Briefly, the SH-SY5Y cells seeded into 96-well plates at a density of 2 × 10^5^ cells/ml for 24 h. The cells were pretreated with different concentrations of Cannabidiol for 24 h followed by incubation with MPP^+^ for another 24 h. The medium was removed and incubated with 20 μl of MTT (5 mg/ml in phosphate buffered saline) for 4 h. Next, added 150 μl DMSO to per well, and the absorbance at 570 nm was measured using a microplate (Thermo Fisher Scientific, United States).

### Immunofluorescence Staining

SH-SY5Y cells were placed in a 12 well plate and treated with or without Cannabidiol, and MPP^+^ for 24 h. The SH-SY5Y cells were fixed with 4% paraformaldehyde for 30 min at room temperature, next, the cells were permeabilization with 0.2% Triton X 100 for 20 min, Then, the cells were blocked with 1% BSA for 1 h and incubated with primary antibody overnight. The next day, After washing with PBS, the cells were incubated with specific secondary antibodies for 1 h, The cellular nuclei were stained with 4’,6-diamidino-2-phenylindole (DAPI) and cells were evaluated using a fluorescence microscope (IX71; Olympus, Miami, FL, United States).

### Statistical Analysis

All data were presented as mean values ± standard deviation (SD), and the results of the experiment are repeated three times. The difference between the two groups were calculated through the *t*-test, Other statistical variances among multigroups were calculated through the One-way analysis of variance (ANOVA). Statistical analyses were performed by GraphPad Prism 8.0 software (GraphPad Software, Inc). *P* < 0.05 was considered to indicate a statistically significant difference.

## Results

### Cannabidiol Alleviated Cytotoxicity in the MPP^+^-Induced SH-SY5Y Cells

**FIGURE 1:**
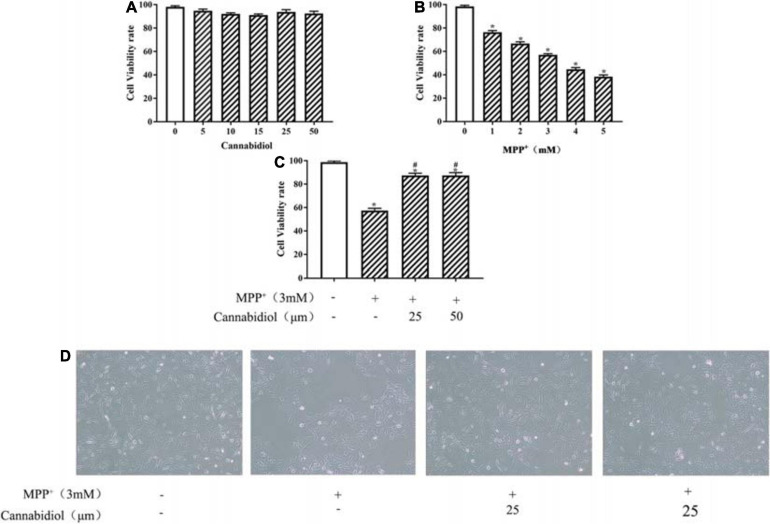
Cannabidiol protected SH-SY5Y cells against MPP^+^-induced cytotoxicity and Loss of Tyrosine Hydroxylase (TH) expression. **(A–C)** SH-SY5Y cells were treated with different concentrations of Cannabidiol or MPP^+^ for 24 h and Cells viability was measured by the 3-(4,5-dimethylthiazol-2-yl)-2,5-diphenyltetrazolium bromide (MTT) assay. **(D)** Cells viability was Observed by Microscopic (100×); The expression of TH in SH-SY5Y cells were measured by western blotting; *p < 0.05, compared with control group, ^#^*p* < 0.05 compared with MPP+ alone.

The viability of SH-SY5Y cells were detected using the MTT assay. The treatment of Cannabidiol (0, 5, 10, 15, 25, and 50 μM) had no significant effect on SH-SY5Y cells viability (Figure 1A). Compared with untreated cell, the treatment of MPP^+^ (0, 1, 2, 3, 4, and 5 mM) significantly reduced SH-SY5Y cells viability (Figure 1B). Based on the MTT results, we chooses 3 mM MPP^+^ used to evaluate the protective effects of Cannabidiol on SH-SY5Y cells. The results showed that treatment with 25 and 50 μM Cannabidiol significantly attenuated MPP^+^-induced loss of SH-SY5Y cells viability (Figures 1C,D). These results suggest that Cannabidiol ameliorated MPP^+^-induced loss of Tyrosine Hydroxylase (TH) expression and cytotoxicity in SH-SY5Y cells.

### Cannabidiol Alleviated Loss of Tyrosine Hydroxylase Expression in the MPP^+^-Induced SH-SY5Y Cells

**FIGURE 2:**
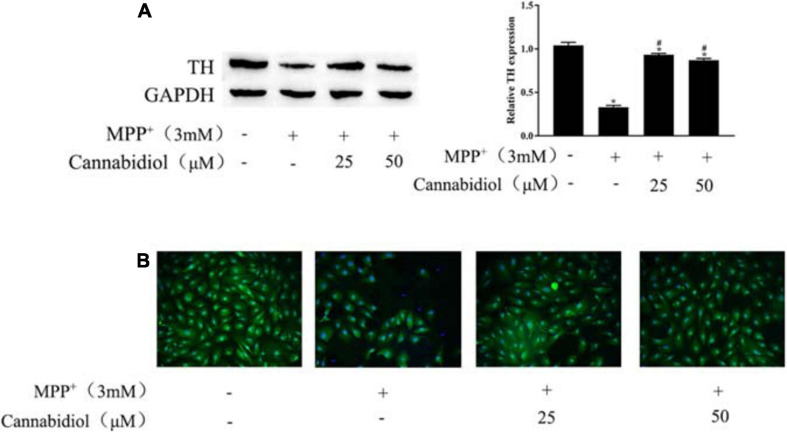
Cannabidiol protected SH-SY5Y cells against MPP^+^-induced Loss of Tyrosine Hydroxylase (TH) expression. **(A)** The expression of TH in SH-SY5Y cells were measured by western blotting; **(B)** The expression of TH in SH-SY5Y cells were measured by Immunofluorescence staining (200×); *p < 0.05, compared with control group, ^#^*p* < 0.05 compared with MPP^+^ alone.

Tyrosine hydroxylase is the rate-limiting enzyme in DA biosynthesis, the decrease in TH expression leads to PD. The TH expression of SH-SY5Y cells were detected using the western blotting and Immunofluorescence staining, the results showed that treatment with 25 and 50 μM Cannabidiol significantly increased TH expression compared with MPP^+^-induced cells (Figures 2A,B). These results suggest that Cannabidiol alleviated loss of TH expression in the MPP^+^-induced SH-SY5Y cells.

### Cannabidiol Alleviated Mitochondrial Dysfunction in MPP^+^-Induced SH-SY5Y Cells

**FIGURE 3:**
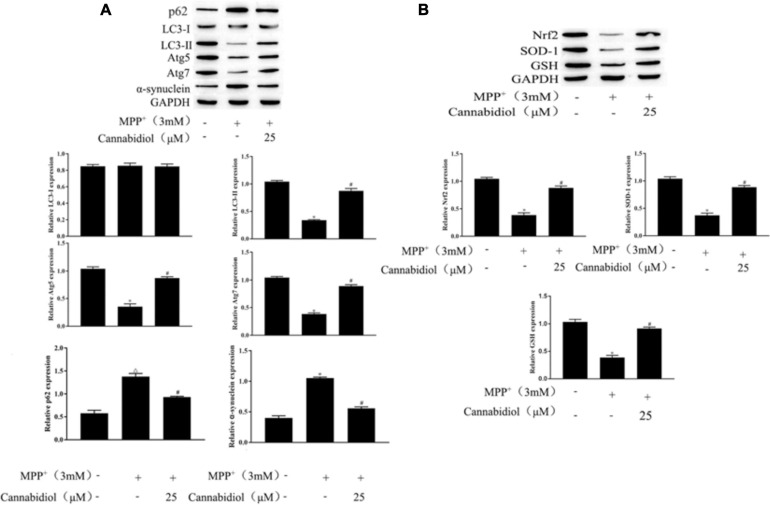
Cannabidiol alleviated mitochondrial dysfunction in MPP^+^-induced SH-SY5Y cells. **(A,B)** The expression of autophagy-related proteins and antioxidant proteins in SH-SY5Y cells were measured by western blotting; *p < 0.05, compared with control group, ^#^*p* < 0.05 compared with MPP^+^ alone.

Having determined that Cannabidiol could alleviated loss of TH expression and cytotoxicity in the MPP^+^-induced SH-SY5Y Cells, we next studied the effect of Cannabidiol on autophagy in cells treated with MPP^+^. The results of western blotting showed that the Cannabidiol significantly enhanced the expression of autophagy-related proteins LC3-II, Atg5/7, decreased the expression p62, and α-synuclein. Simultaneously, pretreatment with Cannabidiol significantly restored the expression of antioxidant proteins Nrf2, SOD-1, and GSH (Figures 3A,B). Thus, Cannabidiol enhanced autophagy in MPP^+^-induced SH-SY5Y cells.

### Blockade of Autophagy and Knockdown of SIRT1 Attenuated the Alleviation Effect of Cannabidiol on the Dysfunction of Mitochondrial Induced by MPP^+^

**FIGURE 4:**
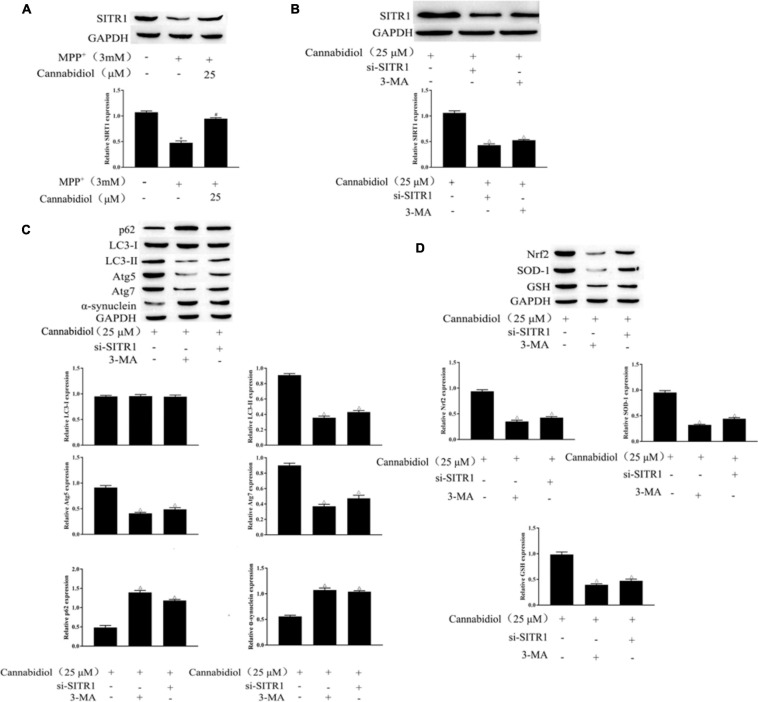
Blockade of autophagy and knockdown of SIRT1 attenuated the alleviation effect of Cannabidiol on the dysfunction of mitochondrial induced by MPP^+^. **(A,B)** The expression of SIRT1 in Cannabidiol treatment SH-SY5Y cells were measured by western blotting; **(C,D)** The expression of autophagy-related proteins and antioxidant proteins in SH-SY5Y cells were measured by western blotting; *p < 0.05, compared with control group, ^#^*p* < 0.05 compared with MPP^+^ alone, ^△^*p* < 0.05 compared with Cannabidiol alone.

SIRT1 is a protein that is downregulated in PD mitochondrial dysfunction and induces autophagy. To find out whether Cannabidiol could induced autophagy in SH-SY5Y Cells by acting on SIRT1, we construct si-SITR1 transfected cells. western blotting showed that SITR1 expression decreased in MPP^+^-induced cells; simultaneously, compared with MPP^+^ induction, Cannabidiol treatment upregulated the expression level of SITR1 (Figures 4A,B). In addition, si-SITR1 transfected and 3-MA (autophagy inhibitor) treatmented decreased the expression of SIRT1 in SH-SY5Y cells compared with the pretreatment of cells with Cannabidiol alone, As shown in Figure 4B. The results of western blotting showed that si-SITR1 transfected and 3-MA treatmented decreased the expression of LC3-II, Atg5/7 and Nrf2, SOD-1, GSH, enhanced the expression p62, α-synuclein, compared with the pretreatment of cells with Cannabidiol alone (Figures 4C,D). This data indicates that blockade of autophagy and knockdown of SIRT1 attenuated the alleviation effect of Cannabidiol on the dysfunction of mitochondrial induced by MPP^+^.

### Cannabidiol Protected SH-SY5Y Cells Mitochondrial Proteins Through SIRT1

**FIGURE 5:**
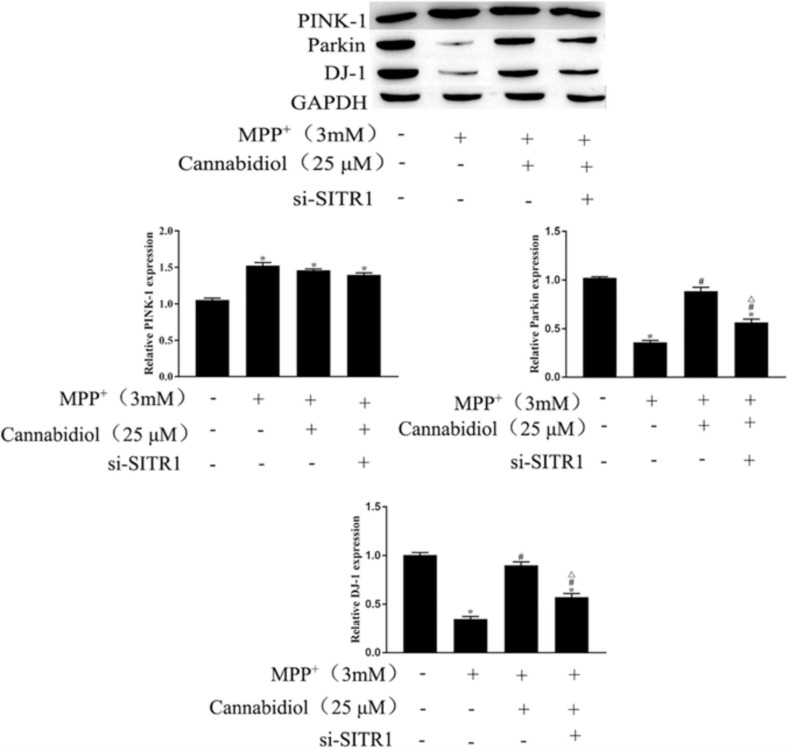
Cannabidiol protected SH-SY5Y cells mitochondrial proteins through SIRT1. The expression of Parkin, PINK-1, DJ-1 in SH-SY5Y cells were measured by western blotting; *p < 0.05, compared with the control group, ^#^*p* < 0.05 compared with MPP^+^ alone, ^△^*p* < 0.05 compared with MPP^+^ + Cannabidiol.

Mitochondrial proteins are destroyed during the development of PD, which may lead to the pathogenesis of PD. We examined the effect of Cannabidiol on the mitophagy regulators Parkin, PINK-1, DJ-1 in MPP^+^-induced SH-SY5Y cells. The results of western blotting showed that MPP^+^ significantly decreased the expression levels of Parkin and DJ-1, compared with untreated cell, as shown in Figure 5. Cannabidiol treatment significantly enhanced the expression levels of Parkin and DJ-1, compared with MPP^+^ alone, as shown in Figure 5. Furthermore, si-SITR1 transfected attenuated the protective effect of Cannabidiol on mitochondrial proteins, as shown in Figure 5. This data indicates that Cannabidiol protected cells mitochondrial proteins through SIRT1.

### Cannabidiol Induced Cell Autophagy by Upregulating SIRT1 to Inhibits NF-κB and NOTCH Pathways

**FIGURE 6:**
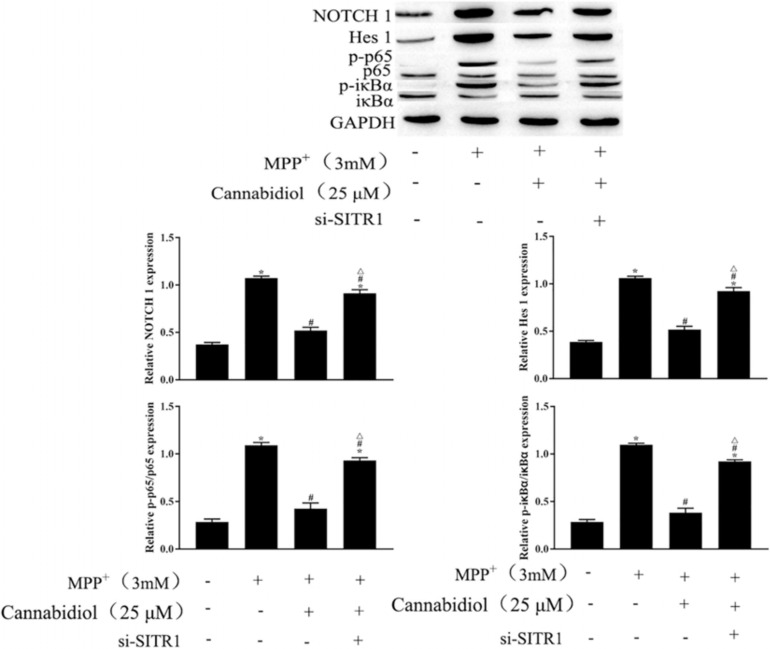
Cannabidiol inhibits NF-κB and NOTCH pathways by upregulating SIRT1. The expression of p65, NOTCH in SH-SY5Y cells were measured by western blotting; ^∗^*p* < 0.05, compared with the control group, ^#^*p* < 0.05 compared with MPP^+^ alone, ^△^*p* < 0.05 compared with MPP^+^ + Cannabidiol.

To further clarify the mechanism of SIRT1 Cannabidiol in PD, we measured the expression levels of signaling molecules involved in the NF-κB and NOTCH pathways. The results of western blotting showed that MPP^+^ significantly enhanced the expression levels of NOTCH1, Hes 1 and phosphorylated p65, iκBα, as shown in Figure 6. Cannabidiol treatment significantly decreased the expression levels of NOTCH1, Hes 1 and phosphorylated p65, iκBα, Furthermore, si-SITR1 transfected attenuated the inhibitory effect of Cannabidiol on inhibitors NF-κB and NOTCH pathways, as shown in Figure 6. Therefore, Cannabidiol inhibits NF-κB and NOTCH pathways by upregulating SIRT1.

## Discussion

The identification of effective protective candidate agents for neurodegenerative diseases is one of the research hotspots in the treatment of PD, Cannabidiol has been reported to treated the psychosis in Parkinson’s disease (Ding et al., 2015; Cassano et al., 2020; Silvestro et al., 2020), However, the neuroprotective potential of Cannabidiol against MPP^+^-mediated PD in SH-SY5Y cells is unclear. In our present study we demonstrated that Cannabidiol induces autophagy to protects SH-SY5Y cells from MPP^+^-mediated mitochondrial dysfunction by upregulating SIRT1 to inhibits NF-κB and NOTCH pathways. We provide evidence that Cannabidiol attenuated the loss of TH and the accumulation of α-synuclein expression, simultaneously restored the levels of antioxidant protein and autophagy protein in SH-SY5Y cells induced by MPP^+^. In addition, STRI1 is highly expressed under the action of Cannabidiol, blockade of autophagy and knockdown of SIRT1 attenuated the alleviation effect of Cannabidiol on the dysfunction of mitochondrial induced by MPP^+^. The current findings reveal that Cannabidiol induces Autophagy to protects SH-SY5Y cells from MPP^+^-mediated mitochondrial dysfunction by upregulating SIRT1 to inhibits NF-κB and NOTCH pathways.

Mitochondrial dysfunction has an integral role in the development of PD (Buneeva et al., 2020; Imam Aliagan et al., 2020), Many studies have reported that MPP^+^-induction is the main source of neuronal cells mitochondrial dysfunction (Xu et al., 2020), MPP^+^-induction causes defects in the activity of the mitochondrial electron transport complex, causes transitions of mitochondrial permeability and increases of oxidative stress, causes death of dopaminergic neurons (Bose and Beal, 2016). A previous study showed that Cannabidiol protects cell survival by improving mitochondrial dysfunction (Vidyadhara et al., 2019). However, the specific mechanism of Cannabidiol in mitochondrial dysfunction remains unclear. In the development of mitochondrial dysfunction, Nrf-2, as an important redox-sensitive transcription factor, reduces cell damage caused by reactive oxygen species by regulating antioxidant proteins (Choi et al., 2017). In the present study, we found that Cannabidiol treatment significantly restored the expression levels of Nrf-2, the most important antioxidant enzyme SOD in the antioxidant system, and the non-enzymatic antioxidant GSH. PINK-1/Parkin pathway is related to mitochondrial damage (Quinn et al., 2020), PINK1 promoted Parkin’s mitochondrial translocation and modification, thus protecting mitochondrial DNA from damage by reactive oxygen species and stimulating the self-repair process of mitochondria (Xu et al., 2020). DJ-1 is a redox sensor of oxidative stress, upregulating DJ-1 antagonize the mitochondrial dysfunction caused by PINK1 mutation, thus, DJ-1 can be used as an important indicator of mitochondrial damage (Lei et al., 2020). In the present study, we found that Cannabidiol pretreatment reduced MPP^+^-mediated mitochondrial damage via activation of PINK-1/parkin and DJ-1.

In the development of PD, the regulation of autophagy is essential (Zhu et al., 2019; Wan et al., 2020). The damage to the autophagy pathway and the resulting accumulation of misfolded α-synuclein and other proteins aggregates represent the common pathobiological characteristics of neurodegenerative diseases such as PD (Erb and Moore, 2020; Kwon et al., 2020). A previous study showed that neurons need autophagy for catabolism to mediate the replacement of damaged organelles and promote synaptic remodeling, thus, autophagy impairment can lead to PD (Moors et al., 2017). In order to deal with the neurological diseases caused by mitochondrial damage, it is necessary to increase the physiological process of mitochondrial autophagy. Previous studies have shown that PINK-1/Parkin pathway is related to mitochondrial damage, Parkin translocation to damaged mitochondria induces mitochondrial autophagy (Quinn et al., 2020). Common antioxidants can reduce oxidative stress damage, but they cannot eliminate the increase of misfolded α-synuclein and other proteins (Yan et al., 2020). Here, we found that Cannabidiol could increase the LC3 levels and decrease the protein expression of p62 and α-synuclein. Combined with the results of Cannabidiol’s effect on oxidative stress, we demonstrated that Cannabidiol can reduce oxidative stress damage, eliminate the increase in misfolded α-synuclein and other proteins, induce autophagy, and alleviate mitochondrial dysfunction.

Mitochondrial biogenesis is a key process in the maintenance of mitochondrial mass, SIRT1 is the main regulator of mitochondrial biogenesis (Mao et al., 2020). The increase of SIRT1 has been considered as a therapeutic strategy for PD, previous research showed that SIRT1 has neuroprotective effects in both *in vivo* and *in vitro* PD models (Li et al., 2020). Simultaneously, SIRT1 was significantly downregulated in neurons treatmented with rotenone or MPP^+^ (Chen et al., 2019). In addition, HA et al. found that Cannabidiol increases the protein expression level of SIRT1 during the activation of white adipocytes (Parray and Yun, 2016). Thus, we speculate that Cannabidiol alleviating PD may be related to the level of SIRT1. In the present study, we found that Cannabidiol significantly increased the expression of SIRT1 in cells, and knockdown of SIRT1 attenuated the alleviation effect of Cannabidiol on the dysfunction of mitochondrial induced by MPP^+^. NF-κB and NOTCH signaling pathway are confirmed to be downstream signaling pathways of SIRT1, SIRT1 could deacetylate the NICD, leading to NOTCH transcriptional inhibition (Bai et al., 2018). Simultaneously, SIRT1 could deacetylate the p65/RELA at lysine 310, leading to NF-κB transcriptional inhibition (Back et al., 2011). Moreover, We also observed that NF-κB and NOTCH signaling pathway participates in the development process of PD (Gan et al., 2020). In this study, we found that Cannabidiol induced cell autophagy by upregulating SIRT1 to inhibits NOTCH1, Hes 1 expression and p65, iκBα phosphorylation.

In summary, the present study showed that Cannabidiol induces autophagy to ameliorate MPP^+^ induced SH-SY5Y cell damage, rescued mitochondrial dysfunction, We further found that Cannabidiol induced cell autophagy by upregulating SIRT1 to inhibits NOTCH and NF-κB pathway. In conclusion, based on our findings, Cannabidiol is a potential candidate for the therapeutic treatment of PD.

## Data Availability Statement

The original contributions presented in the study are included in the article/supplementary material, further inquiries can be directed to the corresponding author/s.

## Ethics Statement

This study was approved by the Ethics Committee of Kunming University of Science and Technology.

## Author Contributions

JXL designed the experiments. SLK and JLL wrote the article. SLK, JLL, and ZHY performed experiments and analyzed data. All the authors read and approved the final manuscript.

## Conflict of Interest

The authors declare that the research was conducted in the absence of any commercial or financial relationships that could be construed as a potential conflict of interest.
